# Evaluating the Onset, Severity, and Recovery of Changes to Smell and Taste Associated With COVID-19 Infection in a Singaporean Population (the COVOSMIA-19 Trial): Protocol for a Prospective Case-Control Study

**DOI:** 10.2196/24797

**Published:** 2020-12-31

**Authors:** Florence Sheen, Vicki Tan, Sumanto Haldar, Sharmila Sengupta, David Allen, Jyoti Somani, Hui Yee Chen, Paul Tambyah, Ciaran G Forde

**Affiliations:** 1 Singapore Institute of Food and Biotechnology Innovation Agency of Science and Research Singapore Singapore; 2 Division of Infectious Diseases Department of Medicine National University Hospital Singapore Singapore; 3 Yong Loo Lin School of Medicine National University Singapore Singapore Singapore; 4 Department of Physiology Yong Loo Lin School of Medicine National University Singapore Singapore Singapore

**Keywords:** SARS-CoV-2, COVID-19, olfactory dysfunction, gustatory dysfunction, anosmia, ageusia, onset, severity, symptom, infectious disease, dysfunction, protocol, marker, recovery, monitoring, taste, smell

## Abstract

**Background:**

Sudden loss of smell and/or taste has been suggested to be an early marker of COVID-19 infection, with most findings based on self-reporting of sensory changes at a single time point.

**Objective:**

To understand the onset, severity, and recovery of sensory changes associated with COVID-19 infection, this study will longitudinally track changes in chemosensory acuity among people with suspected COVID-19 infection using standardized test stimuli that are self-administered over 28 days.

**Methods:**

In a prospective, case-controlled observational study, volunteers will be recruited when they present for COVID-19 screening by respiratory tract polymerase chain reaction test (“swab test”). The volunteers will initially complete a series of questionnaires to record their recent changes in smell and taste ability, followed by a brief standardized smell and taste test. Participants will receive a home-use smell and taste test kit to prospectively complete daily self-assessments of their smell and taste acuity at their place of residence for up to 4 weeks, with all data submitted for collection through web-based software.

**Results:**

This study has been approved by the Domain Specific Review Board of the National Healthcare Group, Singapore, and is funded by the Biomedical Research Council Singapore COVID-19 Research Fund. Recruitment began on July 23, 2020, and will continue through to March 31, 2021. As of October 2, 2020, 69 participants had been recruited.

**Conclusions:**

To our knowledge, this study will be the first to collect longitudinal data on changes to smell and taste sensitivity related to clinically diagnosed COVID-19 infection, confirmed by PCR swab test, in a population-based cohort. The findings will provide temporal insights on the onset, severity, and recovery of sensory changes with COVID-19 infection, the consistency of symptoms, and the frequency of full smell recovery among patients with COVID-19. This self-administered and cost-effective approach has many advantages over self-report questionnaire–based methods and provides a more objective measure of smell and taste changes associated with COVID-19 infection; this will encourage otherwise asymptomatic individuals who are potential spreaders of the virus to self-isolate and seek formal medical diagnosis if they experience a sudden change in sensory acuity. This broadened case finding can potentially help control the COVID-19 pandemic and reduce the emergence of clusters of infections.

**Trial Registration:**

ClinicalTrials.gov NCT04492904; https://clinicaltrials.gov/ct2/show/NCT04492904.

**International Registered Report Identifier (IRRID):**

DERR1-10.2196/24797

## Introduction

Early identification of symptoms associated with SARS-CoV-2, which causes COVID-19, has been recommended to encourage early diagnostic testing and self-isolation and to reduce the risk of community spread of infection. High temperature, continuous dry cough, and fatigue are common clinical symptoms associated with COVID-19 infection; however, numerous recent reports from patients and clinicians worldwide have consistently identified a sudden loss of smell (anosmia) and/or taste (ageusia) as a key early symptom of infection [1-6].

Recent self-report questionnaire data from many countries highlights an association between sudden onset smell and taste loss and COVID-19 infection, with reported incidences of changes in sensory acuity ranging from 11% to >60% [7-13]. A recent systematic review and meta-analysis of studies investigating loss of smell and/or taste with COVID-19 infection reported a pooled prevalence of 52.7% and 43.9% for olfactory and gustatory dysfunction, respectively [14]. Also, Hopkins et al [15] found that 1 in 6 patients reported new onset anosmia as an isolated symptom. The onset of smell and/or taste loss is often abrupt and, unlike other upper respiratory tract infections, it often occurs in the absence of nasal obstruction [13,16]. Importantly, the loss of smell and/or taste occurs early during COVID-19 infection, often before the onset of more established symptoms [8,17-20], and this loss may be an important marker of infection. This finding has prompted many global public health bodies to recommend that individuals who experience sudden changes in sensory acuity should self-isolate and present for diagnostic testing [2-4,21-24].

The majority of published studies investigating smell and taste loss related to COVID-19 have used subjective measurements of smell and taste, specifically self-report questionnaires. In Singapore, there have been anecdotal reports of smell and taste loss with COVID-19 infection [25,26], and in one prospective cohort of patients with COVID-19, it was found that 22.6% experienced acute olfactory loss [27]. The positive predictive value of acute olfactory loss for COVID-19 was 24.1%, and the negative predictive value was 96.5% [27]. Data collected to date have been based largely on self-report questionnaire measures, and there is a lack of objective data on measured smell and taste sensitivity. One study used a validated smell test, the University of Pennsylvania Smell Identification Test (UPSIT), to compare smell acuity in patients diagnosed with COVID-19 with a matched control group; it was shown that 98% of the patient group exhibited some smell dysfunction, scoring significantly lower on the UPSIT compared to controls [28].

In addition, to our knowledge, no longitudinal study has yet been performed to systematically track the onset, severity, and recovery of changes to sensory acuity across the preinfection and postinfection periods of COVID-19, with emerging reports of sustained anosmia among a small proportion of people who have recovered from infection. To date, questionnaire measures alone have been published; these studies relied on self-reporting and may not accurately reflect the true extent of smell and taste changes during COVID-19 infection in the absence of a standardized tool for such measurements. Reliance on self-report questionnaire data and variable approaches could help explain the wide variability in the reported prevalence of olfactory dysfunction with COVID-19 infection (5%-98%) [29]. Therefore, there is a need for objective testing of smell and taste loss in patients infected with COVID-19 using standardized smell and taste stimuli [29-31]. Moreover, due to extensive restrictions on movement, the need to socially distance, and the potential for infection spread with reusable standardized odor and taste test materials, the collection of in-person physical data is challenging. Furthermore, although clinically validated smell and taste assessments are preferred, they are often expensive, and their reuse is not recommended among infected patient populations. Home use sensory tests have been developed to track changes in smell and taste sensitivity using common household items as test stimuli [32,33]. However, these tests rely on participants sourcing and preparing their own test stimuli from available household items; this introduces unwanted stimulus variation, which may present problems for comparability of smell loss across individuals using different stimuli.

The COVOSMIA-19 study will standardize the smell and taste stimuli used by all participants to ensure consistency and provide a disposable, rapid, self-administered home use test, the Singapore Smell and Taste Test (SSTT), to minimize the risk of cross-contamination and infection spread. The trial will enable self-testing for up to 28 consecutive days and will provide a standardized longitudinal assessment of smell and taste function to enable tracking of the onset, severity, and recovery of the sensory changes reported to occur with COVID-19 infection. The standardized smell and taste test will also be compared with self-reported questionnaire measures of smell and taste changes and measured changes to sensory acuity using common household items (an approach previously used by [32]).

The primary objectives of this study are to (1) assess the prevalence of sudden changes in smell and taste sensitivity with COVID-19 infection in a population at risk for COVID-19 infection in Singapore, (2) establish the temporal onset, severity, and recovery of changes in smell and taste sensitivity with COVID-19 infection, and (3) evaluate the efficacy of the SSTT as a rapid, cost-effective, self-administered measure of changes to smell and taste compared to previous measures using household items. The secondary objective of this study is to investigate the effects of loss of smell and taste acuity on food enjoyment and appetite as well as to establish the impact of COVID-19 infection on food-related markers of quality of life. We hypothesize that loss of smell and taste acuity will be associated with COVID-19 infection, will return upon recovery from infection for the majority of patients, and will result in short-term reductions in appetite, food enjoyment, and food-related quality of life.

## Methods

### Study Design

**Figure 1:**
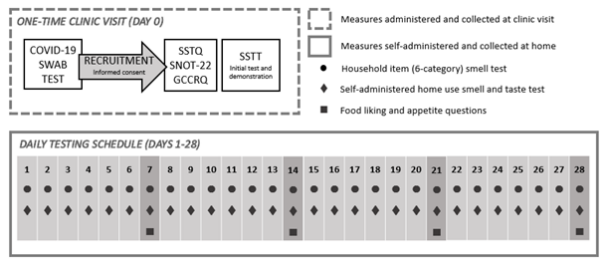
Frequency of the different test measures for the COVOSMIA-19 clinical trial. GCCRQ: Global Consortium for Chemosensory Research Questionnaire; SNOT-22: Sino-Nasal Outcome Test; SSTQ: Singapore Smell and Taste Questionnaire; SSTT: Singapore Smell and Taste Test.

The study will track changes in smell and taste with COVID-19 infection using a prospective longitudinal, case-control study design. Participants will be recruited from volunteers presenting at hospitals and hospital facilities across Singapore for COVID-19 screening. After providing informed consent, the study participants will be asked to report on changes in their smell and taste sensitivity in the previous 2 weeks via a series of questionnaires; they will also be asked to prospectively track daily changes in smell and taste acuity using a self-assessment test and a series of questionnaire measures to measure the onset, severity, and recovery of changes in their chemosensory acuity for the next 28 days. A schematic of the study procedure is shown in Figure 1, and the schedule for enrolment and assessment is shown in Table 1.

**Table 1:** Schedule of participant enrolment, intervention, and assessment in the COVOSMIA-19 trial.

Timepoint		Study period						
		Enrollment	In-clinic	At home (4 weeks)				Closeout
		Day 0	Day 0	Week 1	Week 2	Week 3	Week 4	Day 28
**Enrollment**								
	Eligibility screen	✓						
	Informed consent	✓						
**Procedure**								
	Questionnaires		✓					
	SSTT^a^ testing kit demonstration		✓					
	Initial SSTT session		✓					
	SSTT (daily)^b^			✓	✓	✓	✓	✓
	Additional questions (once per week)			✓	✓	✓	✓	
**Assessments**								
	Baseline variables: gender, age, education status, nationality, ethnicity, employment status, smoking status, vaping status, medication, COVID-19 test result		✓					
	Primary outcome variables: COVID-19 symptoms (and onset), smell and taste acuity			✓	✓	✓	✓	✓
	Secondary outcome variables: appetite, food enjoyment, eating behaviors, nausea, weight, food-related quality of life		✓	✓	✓	✓	✓	✓

^a^SSTT: Singapore Smell and Taste Test.

^b^Participants were asked to complete the SSTT daily from Day 1 to Day 28.

### Recruitment Procedure

Participants will be recruited from patients applying to a hospital and its ancillary units for COVID-19 screening by nasal swab polymerase chain rection (PCR) testing. Following their COVID-19 screening, individuals will be approached and invited to participate in this study and to provide informed consent.

### Inclusion Criteria for Participants

Participants are required to be aged >21 years, reside in Singapore for the next 28 days, possess a mobile device with a 3G or 4G network, and know how to use mobile apps (ie, a quick response [QR] scanner).

### Exclusion Criteria for Participants

Participants will be excluded from the study if they are unable to provide informed consent, are allergic or intolerant to any of the test items (ie, mango fragrance, jasmine fragrance, sugar, salt, coffee powder, or lime powder), are currently pregnant, or do not agree to allow the study team to access their medical records to obtain their COVID-19 infection status (ie, swab test result).

### Ethical Approval and Consent to Participate

The study (ref: 2020/00810) has been approved by the Domain Specific Review Board of the National Healthcare Group, Singapore. Written informed consent will be obtained from all eligible participants prior to entry into the study. Any adverse events or unintended effects of the study procedure will be reported to the ethics committee. This information will be given to participants both verbally and in written form (in their copy of the informed consent form) during recruitment. All data will be anonymized through the use of a unique identification number for each participant and will be stored on password-protected computers. Participants will not receive any monetary compensation for their participation in the study.

Any changes to the above protocol will be reported to the Domain Specific Review Board as appropriate. In addition, these changes will be documented on ClinicalTrials.gov. The results of this study will be disseminated through reports, publication of articles in scientific journals, publication of articles for public dissemination, and conference presentations.

### Measures

On the day of their COVID-19 swab test (Day 0), after consenting to participate in the trial, participants will complete three questionnaires to document recent changes in their smell, taste, and chemesthetic ability and to detail the frequency of a wide range of nasopharyngeal symptoms. These questionnaires include the Singapore Smell and Taste Questionnaire (SSTQ, Multimedia Appendix 1), the 22-item Sino-Nasal Outcome Test (SNOT-22) [34], and the recently developed Global Consortium for Chemosensory Research questionnaire (GCCRQ) [12]. Participants will receive the Singapore Smell and Taste Test (SSTT, Multimedia Appendix 2), a standardized home use testing kit to monitor their smell and taste acuity, and will be given a demonstration of the self-assessment procedure that they will complete daily at their place of residence for the following 28 days. All the questionnaires and instructions are available in English, Mandarin, Tamil, and Bengali, and the participants will complete all the measures via Compusense Cloud web-based data acquisition software (Compusense Inc).

### The SSTQ

Participants will complete the SSTQ (Multimedia Appendix 1) to record any recent changes in smell and taste and to describe the occurrence of sino-nasal or other COVID-19 symptoms. Participants will rate their current smell and taste sensitivity using the SSTQ (Day 0) to provide a baseline measure for their smell and taste sensitivity. This initial self-report questionnaire will quantify any recent changes in smell and taste acuity and includes questions on the participants’ basic demographics, the COVID-19–related symptoms they have experienced, the temporal onset of these symptoms, the smell and taste acuity of the participants, and their appetite-related quality of life (eg, enjoyment of food). See Multimedia Appendix 3 for information on the sources of the questionnaire items.

### The SNOT-22

The SNOT-22 will be used to quantify the presence and severity of nasal disorder symptoms [34]. Participants will be provided with a list of 22 symptoms and social and emotional consequences of nasal disorder, and they will be asked to rate the severity of each problem on a 6-point scale from “no problem” to “problem as bad as it can be.” This measure has been included to assess symptoms that are commonly associated with nasal disorders and to make a distinction between changes in smell and taste due to nasal disorders and congestion and those associated with COVID-19 infection. Current research suggests that smell and taste loss with COVID-19 infection often occurs without nasal obstruction [13,16]; therefore, we expect participants with smell and taste loss to have a differential prevalence of other common nasal disorder symptoms (eg, blocked nose).

### The GCCRQ

The GCCRQ questionnaire was developed and implemented by the Global Consortium for Chemosensory Research to assess whether and how COVID-19 infection affects sense of smell, taste, and chemesthetic sensitivity [12]. Participants are asked about their COVID-19 testing status and symptoms, and they complete 18 items relating to their sense of smell, taste, chemesthetic ability, and food flavors before, during, and after COVID-19 illness. Due to the potential for additional participant burden, the completion of this questionnaire is optional and will not affect the study participation. For participants who complete the GCCRQ, we will ascertain the extent to which the measures obtained using this approach correlate with the measures in the study.

### Daily Smell and Taste Testing

The study participants’ smell and taste acuity will be prospectively measured for up to 28 days following their clinic visit for COVID-19 testing using the home-based assessment tools detailed in this section. At the start of each daily test session, participants are to report on any changes in their smell and taste acuity by selecting either “improved,” “stayed the same,” or “worsened” before rating their current sense of smell and taste from “no sense of [smell/taste] at all” to “extremely strong sense of [smell/taste].” Participants will also be asked to “check all that apply” across a list of symptoms they have experienced in the last 24 hours from a list of COVID-19–relevant symptoms.

### The SSTT

Participants will be provided with their own standardized home-use smell and taste test that they will complete daily for up to 28 consecutive days (Multimedia Appendix 2). The questioning approach is based on that used in the Yale School of Medicine “Jiffy” Test of mell Sensitivity [35], in which respondents perform a similar procedure with Jif peanut butter.

The SSTT smell test includes two odors—a food odor (mango) and a nonfood odor (detergent)—both of which consist of odor mixtures that have been formulated by an international fragrance manufacturer (Symrise AG). The odor mixtures were chosen to avoid possible specific anosmias to individual odor compounds, and the odors are dosed at the suprathreshold level in specialized odor delivery pens (Otto Hut). Both fragrances have been tested to deliver a suprathreshold odor at a consistent intensity and stability for the duration of the home-use test period. To perform the test, the participants remove the pen lid and place the pen 3 inches from their nose while breathing normally. For each smell, participants will identify the smell and rate the perceived intensity on a visual analog scale (VAS) from “not strong at all” to “extremely strong smell.”

For the daily taste test, participants will be provided with small quantities of powder selected to represent the four prototypical tastes: “sweet” (table sugar, NTUC FairPrice), “salty” (table salt, NTUC FairPrice), “bitter” (granulated coffee powder, Nescafé), and “sour” (lime powder, Knorr). The participants will be instructed to take a small amount of each item in a sequential monadic order on the tip of their tongue with a small spoon; they will then be asked to identify and rate the perceived intensity of the taste on a VAS from “not strong at all” to “extremely strong taste” through a web-based questionnaire completed on their smartphone or other device. The participants will be asked to rinse with water between each taste stimulus and will be provided with sufficient taste stimuli for the 28 days of assessment.

### Common Household Item Smell Test

For comparison with the SSTT, participants will be asked to complete a daily assessment of smell using household items (see Multimedia Appendix 2). To assess the participants’ olfactory sensitivity, we will provide them with 6 categories of common household items. Participants will be asked to choose one odor from a drop-down menu for each category and indicate their perception of it on a scale from “absent” to “heightened.” This approach is based on a previously reported self-assessment measure of smell sensitivity using household items [32], with an additional category of stimuli that are particularly high in trigeminal irritation. The items for all categories were adjusted to include culturally appropriate household items that are common in Singapore.

### Additional Questions

Once per week, participants will complete an additional set of questions (Day 7, 14, 21, and 28) relating to their enjoyment of food, appetite, weight, and food-related quality of life (eg, “I no longer enjoy cooking/preparing food”). The goal of this questionnaire is to assess the relative impact of changes in sensory acuity with self-reported appetite and food enjoyment, as it may be related to weight fluctuations observed during the period of infection.

### COVID-19 Diagnosis

During study consent, participants will provide access to the outcome of their COVID-19 diagnostic swab test, which will be associated posthoc with the unique identification number linked to the participant questionnaire and daily home-use test data.

### Sample Size Determination

This prospective trial will compare the onset, severity, and recovery of smell and taste loss as it relates to COVID-19 infection in a case-controlled prospective clinical trial. To perform our proposed cross-sectional analyses (see Statistical Analyses), we require a minimum sample size of 235 based on a power calculation of the sample size required to conduct a logistic regression. This sample will be used to analyze our primary outcome of whether loss of smell and taste acuity predict the likelihood of testing positive for COVID-19 infection. A sample size of 235 is required to observe an effect at 95% power (with a significance level of 0.05) using Pr(Y=1|X=1) H0=0.20 and an odds ratio of 1.723. This odds ratio was calculated as the smallest effect size of interest (SESOI) below which results would not be practically interesting. Given that it is difficult to draw well-informed sample size estimates from the current research, and that we instead opted to use the SESOI to calculate an estimated odds ratio and this minimum sample size, we will also conduct a sensitivity (posthoc) power analysis to demonstrate the effect sizes our final sample size is powered to detect.

### Primary Outcomes

The primary outcomes are (1) to measure the onset, severity, duration, and recovery of changes to smell and taste sensitivity resulting from COVID-19 infection and (2) to assess the efficacy of the SSTT as a simple diagnostic approach. Data will be used to test the association of smell and taste loss with positive COVID-19 infection, measuring changes in sensory acuity from baseline (initial screening, Day 0) to the end of the 4-week monitoring period, to investigate the best predictors of recovery of smell and taste sensitivity. In addition, the primary methodological outcome is a comparison of the consistency and discriminability of self-assessment standardized home use tests compared to both the common household items home use test and the questionnaire-based measures. The findings will inform best practice approaches for future self-assessment of smell and taste acuity among people with suspected COVID-19 infection. This will be measured directly via responses to the daily home-use smell and taste test, complemented by measures from the self-reported questionnaire responses (SSTQ, SNOT-22, GCCRQ).

### Secondary Outcomes

The secondary outcomes will include assessment of experienced symptoms and changes in appetite and food-related quality of life over the duration of smell and taste loss and the 4-week follow-up period. This will be assessed via the home-use test questionnaires.

### Statistical Analysis

Using a case-control study design for people testing positive (case) or negative (control) for COVID-19 infection, data will be collected on changes in smell and taste loss that have occurred in the 2 weeks prior to the COVID-19 test and on smell and taste acuity changes measured on a daily basis for up to 28 days following the COVID-19 test. The data will be used for descriptive comparisons of the onset, duration, and severity of smell and taste changes with COVID-19 infection and to report on the incidence of these changes among participants testing positive for COVID-19 infection (and those recovering from infection).

Logistic regression analysis will be used to estimate the odds ratio for loss of smell and taste sensitivity as a predictor of testing positive for COVID-19 infection. This will also enable comparison of smell and taste loss as a predictor of COVID-19 infection compared to other symptoms, such as fever and dry cough. Pearson correlations will be used to investigate relationships between smell and taste changes and other symptoms. Time-series analysis of variance (ANOVA) models will be run to investigate temporal changes in smell and taste ratings over time, with baseline ratings in smell and taste acuity corrected for at an individual level. Linear mixed models will be used to test for significant differences in smell and taste acuity between groups, with participants stratified by their COVID-19 infection status.

Factor analyses, using oblique rotation and baseline home-use test responses (conducted in-clinic), will be conducted to assess the internal consistency of our smell and taste test. Adjusted odds ratios will be calculated to investigate the best predictors of recovery of smell and taste acuity postinfection. Linear regression will be run to examine whether responses to the smell and taste test predict responses to the household item odor test (smell) and self-reported taste acuity, respectively. Regression analyses will be conducted to investigate the association between loss of smell and taste acuity and self-reported appetite and food-related quality of life. All statistical analyses will be completed using SPSS (IBM Corporation).

### Data Management

Data will be collected on the internet via the CompuSense platform, from which the data will be retrieved and securely stored on a password-protected university computer. These data will be transported into an SPSS file, on which data cleaning and analyses will be conducted.

All participant data will be included in the cross-sectional comparisons, provided the participants have at a minimum undergone COVID-19 swab testing (and we have obtained the result) and completed the SSTQ, SNOT-22, and in-clinic smell and taste test (SSTT). All remote data collection will use web-based forms with forced choice response questionnaires to encourage adherence to the trial protocol, and each participant will receive daily reminders to complete their home-use smell and taste test and questionnaires. Attrition rates are difficult to estimate a priori; however, participants who provide a low number of completed tests (<25%) will be excluded from the longitudinal analyses investigating the home-use testing procedure. Missing data per analysis will be deleted listwise (ie, the individual’s data will be removed from the analysis in question if a single value for any included variable is missing).

### Availability of Data and Materials

No data sets are included in this protocol (not applicable). The study materials (Multimedia Appendices 1-3) are provided. According to National University of Singapore guidelines, it is not possible to grant public access to a participant-level dataset. However, in the event of publication of the trial results, a full protocol, study materials, and any relevant statistical code will be made publicly available on the Open Science Framework.

## Results

The study (ref: 2020/00810) has been approved by the Domain Specific Review Board of the National Healthcare Group, Singapore. This study is funded by the Biomedical Research Council (BMRC) Singapore COVID-19 Research Fund (Project: 12Al04lg1lA04). All study-related materials, tests, stimuli, and procedures are covered by this funding. Recruitment of participants began on July 23, 2020, and will continue through to March 31, 2021. As of October 2, 2020, 69 participants had been recruited into the study.

## Discussion

### Study Overview

This study will longitudinally track the onset, severity, and recovery of changes in chemosensory (smell and taste) acuity among people with and without COVID-19 infection (following swab testing) using standardized assessment tools that can be used in a residential setting. The objectives of this study are to confirm the association between sudden changes in smell and taste sensitivity and COVID-19 infection in an at-risk population in Singapore; to establish the temporal onset, severity, and recovery of changes in smell and taste sensitivity with COVID-19 infection; to evaluate the efficacy of the SSTT as an early diagnostic tool; and to investigate the effects of loss of smell and taste acuity on appetite and food-related quality of life.

Using a longitudinal prospective study design, we aim to establish the association of the onset, severity, and recovery of smell and taste changes with COVID-19 infection while also providing an initial validation of a rapid, cost-effective, self-administered, and standardized approach to tracking spontaneous changes to taste and smell sensitivity in both clinical and residential settings. These findings will enable better identification of asymptomatic or pre-symptomatic carriers of COVID-19 infection, encouraging earlier self-isolation and medical consultation. Currently, a small number of studies have objectively tested smell and taste acuity in patients with COVID-19, with the majority of studies relying on subjective self-report measures [29]. Given that one-time self-report measures may underestimate the prevalence of olfactory impairment [36,37] and fail to capture temporal changes in sensory acuity, the COVISMIA-19 study provides an objective measurement of smell and taste loss in a sample of patients with COVID-19 that can encourage earlier self-isolation and medical consultation. Furthermore, current household item tests of smell and taste acuity are often not standardized and are heavily reliant on stimuli availability and volunteer compliance [32,33]. The COVOSMIA-19 approach will provide a short, easy-to-use, self-administered test approach that standardizes the smell and taste stimuli and can be easily completed by participants in their own home.

### Study Limitations

There is potential for poor compliance by volunteer participants in completing their daily assessments throughout all 28 days of the assessment period. To mitigate this, all questionnaires and home-use test procedures will be short and easy to complete while maintaining the scientific rigor of the test approach. Similarly, the SSTT test measures focus on detecting changes in “usual” sensory perception, and the stimuli have deliberately been selected to monitor changes in suprathreshold perceptual intensity. As such, the SSTT approach will not provide information regarding changes to smell and taste at the perithreshold level (ie, identification, detection discrimination thresholds) or profile odor identification across a wide range of odor stimuli to identify specific anosmias. Although participants will be texted daily reminders to complete their daily home-use tests, their participation is entirely voluntary, with potential for poor adherence and retention. Our proposed procedure for handling potential missing data scenarios are outlined previously (see the Data Management section).

### Study Strengths

A strength of the COVOSMIA-19 study is the longitudinal nature of the participant surveillance and the parallel application of both the questionnaire self-report and standardized test measures for the assessment of loss of smell and taste acuity. Objective measurements of smell and taste acuity are likely to be more reliable [38] and have not yet been used to longitudinally assess smell and taste changes associated with COVID-19 infection. Standardized test stimuli for the home-use test (SSTT) are provided, and longitudinal data collection will be completed remotely via a web-based platform to encourage adherence and facilitate completion of the procedures by the participants in their own home. All test stimuli have been pretested to ensure standardized concentration and stimuli stability and can be used by individuals regularly throughout a period of self-isolation. This approach will both standardize measurement for improved comparison longitudinally and reduce the risk of cross-contamination associated with sharing test measures or attending laboratory testing.

This study will provide new knowledge to increase our understanding of smell and taste losses associated with COVID-19 infection and will evaluate a simple test procedure for future tracking of these changes among individuals with suspected COVID-19 infection form their own home. Through this study, we aim to better identify individuals who suspect COVID-19 infection but are otherwise asymptomatic or have very mild symptoms, encouraging earlier self-isolation and medical consultation.

## Appendix

Multimedia Appendix 1 Singapore Smell and Taste Questionnaire (SSTQ).

Multimedia Appendix 2 Daily smell and taste testing documents.

Multimedia Appendix 3 Table S2. Questionnaire items and their sources.

## Abbreviations

ANOVA: analysis of variance
BMRC: Biomedical Research Council
GCCRQ: Global Consortium for Chemosensory Research questionnaire
PCR: polymerase chain reaction
QR: quick response
SESOI: smallest effect size of interest
SNOT-22: 22-item Sino-Nasal Outcome Test
SSTQ: The Singapore Smell and Taste Questionnaire
SSTT: Singapore Smell and Taste Test
UPSIT: University of Pennsylvania Smell Identification Test
VAS: visual analog scale

## References

[1] Gane S, Kelly C, Hopkins C (2020). Isolated Sudden Onset Anosmia in COVID-19 Infection. A Novel Syndrome?. Rhin.

[2] (2020). Loss of Sense of Smell as Marker of COVID-19 Infection. ENT UK.

[3] Advice for patients with new-onset anosmia during COVID-19 pandemic. ENT UK.

[4] (2020). Anosmia, Hyposmia, and Dysgeusia Symptoms of Coronavirus Disease. American Academy of Otolaryngology.

[5] (2020). Anosmia Alert COVID-19. French Society of ENT.

[6] (2020). Information for Rhinologists on COVID-19. European Rhinologic Society.

[7] Bagheri S, Asghari A, Farhadi M, Shamshiri AR, Kabir Ali, Kamrava SK, Jalessi M, Mohebbi A, Alizadeh R, Honarmand AA, Ghalehbaghi B, Salimi A, Dehghani Firouzabadi F (2020). Coincidence of COVID-19 epidemic and olfactory dysfunction outbreak in Iran. Med J Islam Repub Iran.

[8] Kaye R, Chang CWD, Kazahaya K, Brereton J, Denneny JC (2020). COVID-19 Anosmia Reporting Tool: Initial Findings. Otolaryngol Head Neck Surg.

[9] Klopfenstein T, Kadiane-Oussou N, Toko L, Royer P, Lepiller Q, Gendrin V, Zayet S (2020). Features of anosmia in COVID-19. Med Mal Infect.

[10] Yan CH, Faraji F, Prajapati DP, Boone CE, DeConde AS (2020). Association of chemosensory dysfunction and COVID-19 in patients presenting with influenza-like symptoms. Int Forum Allergy Rhinol.

[11] Lechien JR, Chiesa-Estomba CM, De Siati DR, Horoi M, Le Bon SD, Rodriguez A, Dequanter D, Blecic S, El Afia F, Distinguin L, Chekkoury-Idrissi Y, Hans S, Delgado IL, Calvo-Henriquez C, Lavigne P, Falanga C, Barillari MR, Cammaroto G, Khalife M, Leich P, Souchay C, Rossi C, Journe F, Hsieh J, Edjlali M, Carlier R, Ris L, Lovato A, De Filippis C, Coppee F, Fakhry N, Ayad T, Saussez S (2020). Olfactory and gustatory dysfunctions as a clinical presentation of mild-to-moderate forms of the coronavirus disease (COVID-19): a multicenter European study. Eur Arch Otorhinolaryngol.

[12] Parma V, Ohla K, Veldhuizen M, Niv MY, Kelly CE, Bakke AJ, Cooper KW, Bouysset C, Pirastu N, Dibattista M, Kaur R, Liuzza MT, Pepino MY, Schöpf V, Pereda-Loth V, Olsson SB, Gerkin RC, Rohlfs Domínguez P, Albayay J, Farruggia MC, Bhutani S, Fjaeldstad AW, Kumar R, Menini A, Bensafi M, Sandell M, Konstantinidis I, Di Pizio A, Genovese F, Öztürk L, Thomas-Danguin T, Frasnelli J, Boesveldt S, Saatci O, Saraiva LR, Lin C, Golebiowski J, Hwang LD, Ozdener MH, Guàrdia MD, Laudamiel C, Ritchie M, Havlícek J, Pierron D, Roura E, Navarro M, Nolden AA, Lim J, Whitcroft KL, Colquitt LR, Ferdenzi C, Brindha EV, Altundag A, Macchi A, Nunez-Parra A, Patel ZM, Fiorucci S, Philpott CM, Smith BC, Lundström JN, Mucignat C, Parker JK, van den Brink M, Schmuker M, Fischmeister FPS, Heinbockel T, Shields VDC, Faraji F, Santamaría E, Fredborg WEA, Morini G, Olofsson JK, Jalessi M, Karni N, D'Errico A, Alizadeh R, Pellegrino R, Meyer P, Huart C, Chen B, Soler GM, Alwashahi MK, Welge-Lüssen A, Freiherr J, de Groot JHB, Klein H, Okamoto M, Singh PB, Hsieh JW, Reed DR, Hummel T, Munger SD, Hayes JE, GCCR Group Author (2020). More Than Smell-COVID-19 Is Associated With Severe Impairment of Smell, Taste, and Chemesthesis. Chem Senses.

[13] Lechien JR, Chiesa-Estomba CM, Hans S, Barillari MR, Jouffe L, Saussez S (2020). Loss of Smell and Taste in 2013 European Patients With Mild to Moderate COVID-19. Ann Intern Med.

[14] Tong JY, Wong A, Zhu D, Fastenberg JH, Tham T (2020). The Prevalence of Olfactory and Gustatory Dysfunction in COVID-19 Patients: A Systematic Review and Meta-analysis. Otolaryngol Head Neck Surg.

[15] Hopkins C, Surda P, Kumar N (2020). Presentation of New Onset Anosmia During the COVID-19 Pandemic. Rhin.

[16] Gengler I, Wang JC, Speth MM, Sedaghat AR (2020). Sinonasal pathophysiology of SARS-CoV-2 and COVID-19: A systematic review of the current evidence. Laryngoscope Investig Otolaryngol.

[17] Iacobucci G (2020). Sixty seconds on . . . anosmia. BMJ.

[18] Vukkadala N, Qian ZJ, Holsinger FC, Patel ZM, Rosenthal E (2020). COVID-19 and the Otolaryngologist: Preliminary Evidence-Based Review. Laryngoscope.

[19] Haehner A, Draf J, Dräger S, de With K, Hummel T (2020). Predictive Value of Sudden Olfactory Loss in the Diagnosis of COVID-19. ORL J Otorhinolaryngol Relat Spec.

[20] Lee Y, Min P, Lee S, Kim S (2020). Prevalence and Duration of Acute Loss of Smell or Taste in COVID-19 Patients. J Korean Med Sci.

[21] (2020). Coronavirus - Symptoms. World Health Organization.

[22] (2020). Symptoms of Coronavirus. US Centers for Disease Control and Prevention.

[23] (2020). Updates on COVID-19 (Coronavirus Disease 2019) Local Situation. Singapore Ministry of Health.

[24] (2020). Guidance: COVID-19: epidemiology, virology and clinical features. Public Health England.

[25] Mohan M (2020). ‘We didn’t know there was a virus inside of us’: A young couple’s fight against COVID-19. CNA.

[26] Goh T (2020). I can't smell the orange: NUS don tested positive for Covid-19 after losing sense of smell. The Straits Times.

[27] Chua AJ, Charn TC, Chan EC, Loh J (2020). Acute Olfactory Loss Is Specific for COVID-19 at the Emergency Department. Ann Emerg Med.

[28] Moein ST, Hashemian SM, Mansourafshar B, Khorram-Tousi Ali, Tabarsi P, Doty RL (2020). Smell dysfunction: a biomarker for COVID-19. Int Forum Allergy Rhinol.

[29] Hannum M, Ramirez V, Lipson S, Herriman RD, Toskala AK, Lin C, Joseph PV, Reed DR (2020). Objective Sensory Testing Methods Reveal a Higher Prevalence of Olfactory Loss in COVID-19-Positive Patients Compared to Subjective Methods: A Systematic Review and Meta-Analysis. Chem Senses.

[30] Pellegrino R, Cooper K, Di Pizio A, Joseph PV, Bhutani S, Parma V (2020). Corona Viruses and the Chemical Senses: Past, Present, and Future. Chem Senses.

[31] Menni C, Valdes A, Freidin MB, Sudre CH, Nguyen LH, Drew DA, Ganesh S, Varsavsky T, Cardoso MJ, El-Sayed Moustafa JS, Visconti A, Hysi P, Bowyer RCE, Mangino M, Falchi M, Wolf J, Ourselin S, Chan AT, Steves CJ, Spector TD (2020). Real-time tracking of self-reported symptoms to predict potential COVID-19. Nat Med.

[32] Iravani B, Arshamian A, Ravia A, Mishor E, Snitz K, Shushan S, Roth Y, Perl O, Honigstein D, Weissgross R, Karagach S, Ernst G, Okamoto M, Mainen Z, Monteleone E, Dinnella C, Spinelli S, Mariño-Sánchez F, Ferdenzi C, Smeets M, Touhara K, Bensafi M, Hummel T, Sobel N, Lundström JN (2020). Relationship between odor intensity estimates and COVID-19 prevalence prediction in a Swedish population. Chem Senses.

[33] Vaira LA, Salzano G, Petrocelli M, Deiana G, Salzano FA, De Riu G (2020). Validation of a self-administered olfactory and gustatory test for the remotely evaluation of COVID-19 patients in home quarantine. Head Neck.

[34] Hopkins C, Gillett S, Slack R, Lund V, Browne J (2009). Psychometric validity of the 22-item Sinonasal Outcome Test. Clin Otolaryngol.

[35] (2020). Jiffy Test of Smell Sensitivity. Yale School of Medicine.

[36] Smith W, Murphy C (2009). Epidemiological studies of smell: discussion and perspectives. Ann N Y Acad Sci.

[37] Yang J, Pinto JM (2016). The Epidemiology of Olfactory Disorders. Curr Otorhinolaryngol Rep.

[38] Lechien JR, Cabaraux P, Chiesa-Estomba CM, Khalife M, Hans S, Calvo-Henriquez C, Martiny D, Journe F, Sowerby L, Saussez S (2020). Objective olfactory evaluation of self-reported loss of smell in a case series of 86 COVID-19 patients. Head Neck.

