# Supplementary material for: Evaluating the Onset, Severity, and Recovery of Changes to Smell and Taste Associated With COVID-19 Infection in a Singaporean Population (the COVOSMIA-19 Trial): Protocol for a Prospective Case-Control Study
Source: JMIR Res Protoc. 2020 Dec 31;9(12):e24797. doi: 10.2196/24797 (PMC7781589; doi:10.2196/24797)
Supplement: Multimedia Appendix 1 [file resprot_v9i12e24797_app1.pdf]

Singapore Smell and Taste Questionnaire (SSTQ)

You have been invited to participate in this study as you are currently being screened for COVID-19.

Thank you for volunteering and taking time to complete all the questionnaires in this session.

Gender:
☐ Male
☐ Female
☐ Other not listed here
☐ Prefer not to say

Age (Please enter a number):
[ ]

Highest Education Status:
☐ No formal qualification
☐ Secondary School
☐ High School
☐ Diploma/Degree
☐ Post-graduate qualification

Nationality:
☐ Singaporean
☐ Malay
☐ Chinese
☐ Indian
☐ Bangladeshi
☐ Other (please specify): [ ]

Ethnicity:
☐ Chinese
☐ Malay
☐ Indian
☐ Bangladeshi
☐ Other (please specify): [ ]

What is your employment status in Singapore?
☐ Singaporean citizen – Full Time Employment
☐ Singaporean citizen – Part Time Employment
☐ Singaporean citizen – Unemployed
☐ Singaporean permanent resident – Full Time Employment
☐ Singaporean permanent resident – Part Time Employment
☐ Singaporean permanent resident – Unemployed
☐ S-pass holder – Full Time Employment
☐ Employment Pass
☐ Dependent Pass

Do you smoke?
☐ Yes
☐ Not currently, but I have in the past
☐ No, I have never smoked

Do you vape?
☐ Yes
☐ Not currently, but I have in the past
☐ No, I have never vaped

Singapore Smell and Taste Questionnaire (SSTQ)

Are you currently on any medication(s)?

□ Yes (please specify) [ ]
□ No

Do you have any medical conditions? For example, diabetes, high blood pressure, lung or heart conditions, etc. If you do not have any medical conditions, please put '*none*'.

[ ]

Do you currently have any of the following symptoms? Please select all that apply.

□ Smell loss (total)

□ Smell loss (partial)

□ Diminished taste

□ No other symptoms

□ High temperature (fever)

□ Chills

□ Dry cough

□ Cough with mucus

□ Nasal congestion

□ Runny nose

□ Difficulty breathing

□ Sore throat

□ Muscle aches

□ Fatigue

□ Diarrhoea

□ Abdominal pain

□ Headache

□ Eye itchiness

□ Vomiting/Nausea

□ Other (Please Specify): [ ]

When did you first experience any of the above symptoms?

If you cannot remember the exact date, please put the earliest date you can recall having symptoms.

[Question opens onto a calendar, from which the participants can select a day, month and year]

Did you experience a loss of sense of smell (anosmia) recently?

□ Yes

□ No

~~~~~~~~~SKIP LOGIC~~~~~~~~~

YES:

When did you experience a loss of sense of smell (anosmia) recently?

If you cannot remember the exact date, please put the earliest date you can recall experiencing this.

[Question opens onto a calendar, from which the participants can select a day, month and year]

Please rate the time of onset of loss of sense of smell (in relation to other symptoms):

☐ Only symptom

☐ Prior to other symptoms

☐ Concomitant with other symptoms

☐ After other symptoms

NO:

Skip to following question.

Did you experience a loss of sense of taste (dysgeusia) recently?

☐ Yes

☐ No

~~~~~~~~~SKIP LOGIC~~~~~~~~~

YES:

When did you experience a loss of sense of taste (dysgeusia) recently?

If you cannot remember the exact date, please put the earliest date you can recall experiencing this.

[Question opens onto a calendar, from which the participants can select a day, month and year]

Please rate the time of onset of loss of sense of taste (in relation to other symptoms):

☐ Only symptom

☐ Prior to other symptoms

☐ Concomitant with other symptoms

☐ After other symptoms

NO:

Skip to following question.

Did you have any other symptoms BEFORE the development of smell or taste loss?

- ☐ Yes
- ☐ No

~~~~~~~~SKIP LOGIC~~~~~~~~

YES:

What symptoms did you have BEFORE the development of smell or taste loss?

Please select all that apply.

- ☐ No other symptoms
- ☐ High temperature (fever)
- ☐ Chills
- ☐ Dry cough
- ☐ Cough with mucus
- ☐ Nasal congestion
- ☐ Runny nose
- ☐ Difficulty breathing
- ☐ Sore throat
- ☐ Muscle aches
- ☐ Fatigue
- ☐ Diarrhoea
- ☐ Abdominal pain
- ☐ Headache
- ☐ Eye itchiness
- ☐ Vomiting/Nausea
- ☐ Other (Please Specify): ________

NO:

Skip to following question.

Singapore Smell and Taste Questionnaire (SSTQ)

What symptoms did you have AT THE TIME of smell or taste loss?

Please select all that apply.

☐ No other symptoms

☐ High temperature (fever)

☐ Chills

☐ Dry cough

☐ Cough with mucus

☐ Nasal congestion

☐ Runny nose

☐ Difficulty breathing

☐ Sore throat

☐ Muscle aches

☐ Fatigue

☐ Diarrhoea

☐ Abdominal pain

☐ Headache

☐ Eye itchiness

☐ Vomiting/Nausea

☐ Other (Please Specify): ____________

During the past few days or weeks, have you had a problem with your ability to smell, such as not being able to smell things or things not smelling the way they are supposed to?

☐ Yes

☐ No

~~~~~~~~SKIP LOGIC~~~~~~~~

YES:

How long ago did you first notice a problem with, or a change in, your ability to smell?

☐ Within the past week
☐ About a week ago
☐ 2 weeks ago
☐ 3 weeks ago, or longer

NO:

Skip to following question.

Singapore Smell and Taste Questionnaire (SSTQ)

1. **Please rate your ability to smell, BEFORE your illness:**

NO SENSE OF SMELL AT ALL ——— EXCELLENT SENSE OF SMELL .

2. **Please rate your ability to smell, DURING your illness:**

NO SENSE OF SMELL AT ALL ——— EXCELLENT SENSE OF SMELL .

3. **Please rate your ability to smell, AFTER your illness:**

(Please skip this question if you have not yet recovered)

NO SENSE OF SMELL AT ALL ——— EXCELLENT SENSE OF SMELL

4. **How blocked was your nose, BEFORE your illness:**

COMPLETELY BLOCKED ——— COMPLETELY CLEAR

5. **How blocked was your nose, DURING your illness:**

COMPLETELY BLOCKED ——— COMPLETELY CLEAR

6. **How blocked was your nose, AFTER your illness:**

(Please skip this question if you have not yet recovered)

COMPLETELY BLOCKED ——— COMPLETELY CLEAR

7. **Since becoming ill, does your sense of smell fluctuate?**

☐ No – it is always the same
☐ Yes – it comes and goes
☐ Don’t know

Singapore Smell and Taste Questionnaire (SSTQ)

~~~~~~~~SKIP LOGIC~~~~~~~~

YES:

Since becoming ill ...

- ☐ Smells smell less strong than they did before
- ☐ Smells smell different compared to before being ill (i.e. the quality of smell has changed).
- ☐ I can smell things that aren't there (e.g. I can smell random burning when nothing is on fire).
- ☐ Other (please specify) ______

NO:

Skip to following question.

Please rate your sense of smell right now.

NO SENSE OF SMELL AT ALL |————————————————| EXTREMELY STRONG SENSE OF SMELL

During the past few days or weeks, have you had a problem with your ability to taste, such as not being able to taste things or things not tasting the way they are supposed to?

☐ Yes

☐ No

~~~~~~~~SKIP LOGIC~~~~~~~~

YES:

How long ago did you first notice a problem with, or a change in, your ability to taste?

☐ Within the past week
☐ About a week ago
☐ 2 weeks ago
☐ 3 weeks ago, or longer

NO:

Skip to following question.

Singapore Smell and Taste Questionnaire (SSTQ)

1. **Please rate your ability to taste BEFORE your illness:**

NO SENSE OF TASTE AT ALL | ———————————— | EXCELLENT SENSE OF TASTE

2. **Please rate your ability to taste DURING your illness:**

NO SENSE OF TASTE AT ALL | ———————————— | EXCELLENT SENSE OF TASTE

3. **Please rate your ability to taste AFTER your illness:**

   (Please skip this question if you have not yet recovered)

NO SENSE OF TASTE AT ALL | ———————————— | EXCELLENT SENSE OF TASTE

Did you experience any other changes to tastes?

☐ Yes (Please specify): [ ]

☐ No

~~~~~~~~SKIP LOGIC~~~~~~~~

YES

Which of the following tastes have been affected?

(Please check all that apply)

☐ Sweet
☐ Salty
☐ Sour
☐ Bitter
☐ None – I can taste all of the above

NO:

Skip to following question.

Please rate your sense of taste right now:

NO SENSE OF TASTE AT ALL | ———————————— | EXTREMELY STRONG SENSE OF TASTE

Singapore Smell and Taste Questionnaire (SSTQ)

Since your taste or smell problem began, your appetite has been:
☐ I do not have a taste or smell problem
☐ Better
☐ Unchanged
☐ Worse

Since your taste or smell problem began, you have enjoyed food:
☐ I do not have a taste or smell problem
☐ More
☐ The same
☐ Less

Does your taste or smell problem affect the way you eat (e.g., types of foods, meal, and snacking frequency)?
☐ I do not have a taste or smell problem
☐ No
☐ Yes (Describe Change): ______

Since your taste or smell problem began, have you altered the amount of salt, sugar or spices you add to your food?
☐ I do not have a taste or smell problem
☐ No
☐ Yes (Describe Change): ______

Since your taste or smell problem began, have you started to strongly dislike or avoid certain foods?
☐ I do not have a taste or smell problem
☐ No
☐ Yes (Specify foods): ______

Since your taste or smell problem began, have you had a strong desire or craving for certain foods?
☐ I do not have a taste or smell problem
☐ No
☐ Yes (Specify foods): ______

Do you feel nauseated or sick to your stomach often?
☐ No
☐ Yes

~~~~~~~~SKIP LOGIC~~~~~~~~

YES

How severely?
☐ Mildly
☐ Extremely
When?
______

Singapore Smell and Taste Questionnaire (SSTQ)

NO:

Skip to following question.

Since your taste or smell problem began, your weight has:

o I do not have a taste/smell problem
o Increased (Please specify change (kg)): ______
o Remained unchanged
o Decreased (Please specify change (kg)): ______

~~~~~~~~SKIP LOGIC~~~~~~~~

Increased/decreased

Was the change in your weight due to your taste or smell problem?

o I have not experienced a weight change
o No (Specify cause): ______
o Yes (Specify cause): ______

Remained unchanged/I do not have a taste/smell problem:

Skip to following question.

Your current weight is ______ **(kg)**
Your current height is ______ **(m)**

Because of my loss of smell/taste…

	STRONGLY DISAGREE	DISAGREE	DISAGREE SOMEWHAT	NEITHER AGREE NOR DISAGREE	AGREE SOMEWHAT	AGREE	STRONGLY AGREE
I no longer enjoy the taste of my food							
I no longer enjoy							

**the smell of my food**							
**I no longer enjoy cooking /preparing food**							
**I no longer enjoy eating food**							
**I find it difficult to eat food**							
**I find meals boring**							

If there is anything else you would like to tell us about how your loss of sense of smell and taste has affected you or your eating habits, please use the space below:

Please select all the symptoms you have experienced in the last 24 hours:

☐ Smell loss (total)

☐ Smell loss (partial)

☐ Diminished taste

☐ No other symptoms

☐ High temperature (fever)

☐ Chills

☐ Dry cough

☐ Cough with mucus

☐ Nasal congestion

☐ Runny nose

☐ Difficulty breathing

☐ Sore throat

☐ Muscle aches

☐ Fatigue

☐ Diarrhoea

☐ Abdominal pain

☐ Headache

☐ Eye itchiness

☐ Vomiting/Nausea

☐ Other (Please Specify): [ ]

Do you know your COVID-19 status?

☐ Positive (as confirmed by test)
☐ Test results pending
☐ Presumed positive (as determined by medical provider; no test)
☐ Test negative
☐ Other (Please Specify): [ ]

Is there anything else you wish to share?

………
………
………
………
…………………………………………
