# Supplementary material for: Evaluating the Onset, Severity, and Recovery of Changes to Smell and Taste Associated With COVID-19 Infection in a Singaporean Population (the COVOSMIA-19 Trial): Protocol for a Prospective Case-Control Study
Source: JMIR Res Protoc. 2020 Dec 31;9(12):e24797. doi: 10.2196/24797 (PMC7781589; doi:10.2196/24797)
Supplement: Multimedia Appendix 2 [file resprot_v9i12e24797_app2.pdf]

Additional File 3: Daily smell and taste testing documents

Daily Home-Use Test Instructions – page 2

Daily Testing Questionnaire – pages 3-9

Additional Questions – pages 10-13

Daily Home-use Test & Follow-up Questionnaire

Please remember to complete the Test & Questionnaire at the same time every day

Please follow the instructions below to complete your daily self-reported questionnaire.

1.

Please scan the QR code. This will bring you to the login page.

请扫描二维码（QR code）。二维码会连接到登入网址。

কিউআর কোডটি স্ক্যান করুন এটি আপনাকে লগইন পৃষ্ঠায় নিয়ে আসবে।

QR குறியீட்டை ஸ்கேன் செய்யுங்கள். இது உங்களை உள்நுழைவு பக்கத்திற்கு கொண்டு வரும்.

2.

Username & Password:

SCCTXXXX

You may login using the login information above.

您可以按照以上的用户名和密码来登入网站。

আপনি উপরের লগইন তথ্য ব্যবহার করে লগইন করতে পারেন।

மேலே உள்ள உள்நுழைவு தகவலைப் பயன்படுத்தி நீங்கள் உள்நுழையலாம்.

3.

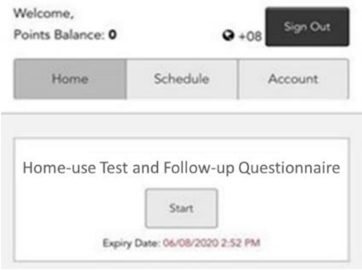

Please select '**Home-use Test and Follow-up Questionnaire**'.

请选择'**Home-use Test and Follow-up Questionnaire**'。

আজকের স্ব-প্রতিবেদনিত প্রশ্নাবলী সম্পূর্ণ করতে দয়া করে '**Home-use Test and Follow-up Questionnaire**' নির্বাচন করুন

'**Home-use Test and Follow-up Questionnaire**' என்பதைத் தேர்ந்தெடுக்கவும்.

Teaspoon / 茶匙/ চা চামচ/ டீஸ்பூன்

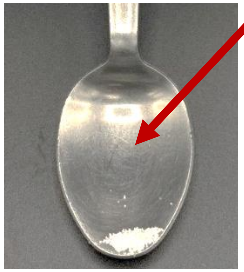

Sugar/ 糖/ চিনি/ சர்க்கரை

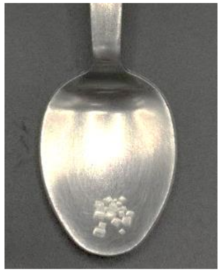

Salt/ 盐/ লবণ/ உப்பு

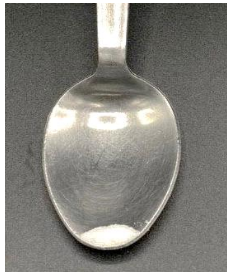

Lime Powder/ 酸酐粉/ লেবু গুঁড়ো / எலுமிச்சை பொடி

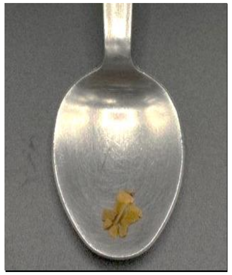

Coffee Powder/咖啡粉/ কফি পাউডার/ காபி பொடியை

Please attach Odour **Pen 1** here

Please attach Odour **Pen 2** here

Thank you [ID] for taking time to complete the self-reported questionnaire!

This questionnaire will be completed over a total of 28 days.

This is today's Self-Reported Questionnaire.

Since the last test, my sense of smell has…

- Improved
- Stayed the same
- Worsened

Please rate your sense of smell right now:

NO SENSE OF SMELL AT ALL —————————— EXTREMELY STRONG SENSE OF SMELL

Since the last test, my sense of taste has…

- Improved
- Stayed the same
- Worsened

Please rate your sense of taste right now:

NO SENSE OF TASTE AT ALL —————————— EXTREMELY STRONG SENSE OF TASTE

For <u>each</u> of the following categories of odour, <u>please select 1 odour</u> and indicate on the scale your perception of it.

<u>Category 1:</u>

Drop-down menu showing the options:
Orange
Apple
Watermelon
Papaya
Mango
Banana
Pineapple
Fruit Jam

ABSENT —————— NORMAL —————— HEIGHTENED

Category 2:

Drop-down menu showing the options:
Hair shampoo
Body wash
Hand soap
Lavender
Air freshener
Bleach
Sunscreen
Perfume

ABSENT NORMAL HEIGHTENED

Category 3:

Drop-down menu showing the options:
Chicken rice
Soy sauce
Curry gravy
Coconut
Cheese
Sesame oil

ABSENT NORMAL HEIGHTENED

Category 4:

Drop-down menu showing the options:
Pandan leaves
Coriander
Onion
Chocolate
Ketchup
Coffee
Tea
Cola

ABSENT NORMAL HEIGHTENED

Category 5:

Drop-down menu showing the options:
Cumin
Five spice
Lemon grass
Ginger
Chilli
Garlic
Sambal
Cardamom
Cinnamon

ABSENT NORMAL HEIGHTENED

Category 6:

Drop-down menu showing the options:
Vinegar
Toothpaste/menthol
Mustard
Wasabi
Cloves
Vodka/Scotch

ABSENT NORMAL HEIGHTENED

The kind of items that I cannot smell or taste normally are:

☐ I cannot smell/taste anything
☐ Foods/beverages (specify)
☐ Tobacco products (specify)
☐ Detergent/washing-up liquid/shampoo (specify)
☐ All of the above
☐ Other (specify)
☐ I can smell/taste everything

You will now complete the smell test.

Please use your sniffing pens (2) to answer the subsequent questions.

Please take the <u>first pen</u> and open it.

Bring the pen about 3 inches from your nose (approximately the width of your hand).

Please sniff.

Do you smell it?

- Yes. The smell is ☐
- No

The smell is…

NOT STRONG AT ALL |————————————————| EXTREMELY STRONG SMELL

Please take the <u>second pen</u> and open it.

Bring the pen about 3 inches from your nose (approximately the width of your hand).

Please sniff.

Do you smell it?

- Yes. The smell is ☐
- No

The smell is…

NOT STRONG AT ALL |————————————————| EXTREMELY STRONG SMELL

You will now complete the taste test.

Please use the taste test items (4) to answer the subsequent questions.

Please take a small amount of <u>salt</u> to the tip end of your tongue with a small spoon to taste.

Do you taste it?

- Yes
- No

The taste is…

NOT STRONG AT ALL	———————————	EXTREMELY STRONG SMELL

Please take a small amount of <u>sugar</u> to the tip end of your tongue with a small spoon to taste.

Do you taste it?

- Yes
- No

The taste is…

NOT STRONG AT ALL	———————————	EXTREMELY STRONG SMELL

Please take a small amount of <u>sour plum powder</u> to the tip end of your tongue with a small spoon to taste.

Do you taste it?

- Yes
- No

The taste is…

NOT STRONG AT ALL	———————————	EXTREMELY STRONG SMELL

Please take a small amount of <u>coffee powder</u> to the tip end of your tongue with a small spoon to taste.

Do you taste it?

· Yes

· No

The taste is…

NOT STRONG AT ALL ——————————————— EXTREMELY STRONG SMELL

Please select all the symptoms you have experienced in the last 24 hours:

Please tick all that apply.

☐ Smell loss (total)
☐ Smell loss (partial)
☐ Diminished taste
☐ No other symptoms
☐ High temperature (fever)
☐ Chills
☐ Dry cough
☐ Cough with mucus
☐ Nasal congestion
☐ Runny nose
☐ Difficulty breathing
☐ Sore throat
☐ Muscle aches
☐ Fatigue
☐ Diarrhoea
☐ Abdominal pain
☐ Headache
☐ Eye itchiness
☐ Vomiting/Nausea
☐ Other (Please Specify): ______

Is there anything else you wish to share?

………
………
………

....

Thank you for completing Day [X]'s self-reported questionnaire.

Please come back again, same time, tomorrow to complete the questionnaire again.

You may click *'Pause'* below to exit.

Since your taste or smell problem began, your appetite has been:

 I do not have a taste or smell problem
 Better
 Unchanged
 Worse

Since your taste or smell problem began, you have enjoyed food:
 I do not have a taste or smell problem
 More
 The same
 Less

Does your taste or smell problem affect the way you eat (e.g., types of foods, meal, and snacking frequency)?
 I do not have a taste or smell problem
 No
 Yes (Describe Change):

Since your taste or smell problem began, have you altered the amount of salt, sugar or spices you add to your food?
 I do not have a taste or smell problem
 No
 Yes (Describe Change):

Since your taste or smell problem began, have you started to strongly dislike or avoid certain foods?
 I do not have a taste or smell problem
 No
 Yes (Specify foods):

Since your taste or smell problem began, have you had a strong desire or craving for certain foods?
 I do not have a taste or smell problem
 No
 Yes (Specify foods):

Do you feel nauseated or sick to your stomach often?
☐ No
☐ Yes

~~~~~~~~SKIP LOGIC~~~~~~~~

YES

How severely?
☐ Mildly
☐ Extremely
When?

NO:

Skip to following question.

Since your taste or smell problem began, your weight has:
o I do not have a taste/smell problem
o Increased (Please specify change (kg)):
o Remained unchanged
o Decreased (Please specify change (kg)):

~~~~~~~~SKIP LOGIC~~~~~~~~

Increased/Decreased:

Was the change in your weight due to your taste or smell problem?

o I have not experienced a weight change
o No (Specify cause):
o Yes (Specify cause):

Remained unchanged/I do not have a taste/smell problem:

Skip to following question.

Your current weight is **(kg)**
Your current height is **(m)**

Because of my loss of smell/taste…

	STRONGLY DISAGREE	DISAGREE	DISAGREE SOMEWHAT	NEITHER AGREE NOR DISAGREE	AGREE SOMEWHAT	AGREE	STRONGLY AGREE
I no longer enjoy the taste of my food							
I no longer enjoy the smell of my food							
I no longer enjoy cooking /preparing food							
I no longer enjoy eating food							
I find it difficult to eat food							
I find meals boring							

Have you consulted your GP/another healthcare professional about your loss of sense of smell/taste?
☐ Yes
☐ No

~~~~~~~~SKIP LOGIC~~~~~~~~

YES:

Did this lead to any treatment for the loss of sense of smell/taste?
☐ Yes
☐ No

Did you receive any medication prescription?

☐ Yes (Please specify): ____________
☐ No

NO:

Skip to following question.

Have you sought advice from other platforms (e.g. social media, charities)?

☐ Yes (Please specify): [ ]
☐ No

If there is anything else you would like to tell us about how your loss of sense of smell and taste has affected you or your eating habits, please use the space below:

……
……
……
……
………………………………………
