# Supplementary material for: Evaluating the Onset, Severity, and Recovery of Changes to Smell and Taste Associated With COVID-19 Infection in a Singaporean Population (the COVOSMIA-19 Trial): Protocol for a Prospective Case-Control Study
Source: JMIR Res Protoc. 2020 Dec 31;9(12):e24797. doi: 10.2196/24797 (PMC7781589; doi:10.2196/24797)
Supplement: Multimedia Appendix 3 [file resprot_v9i12e24797_app3.pdf]

Table S2. Questionnaire items and their source

QUESTIONNAIRE	***QUESTION CONTENT***	***RELEVANCE***	***SOURCE***
SSTQ	Gender	Demographics	Self-devised
SSTQ	Age	Demographics	Self-devised
SSTQ	Highest education status	Demographics	Self-devised
SSTQ	Nationality	Demographics	Self-devised
SSTQ	Ethnicity	Demographics	Self-devised
SSTQ	Singapore employment status	Demographics	Self-devised
SSTQ	Smoking status	Demographics	Self-devised
SSTQ	Vaping status	Demographics	Self-devised
SSTQ	Medication (Y/N)	General health	Self-devised
SSTQ	Medical condition(s)	General health	GCCR [1]
SSTQ	Symptoms (list) [a]	COVID-19	Columbia University Irving Medical Centre COVID-19 symptom assessment/ GCCR [1]
SSTQ	First experience of symptoms	COVID-19	Self-devised
SSTQ	Anosmia (Y/N)	Smell	Self-devised
SSTQ	Anosmia (when?)	Smell	American Academy of Otolaryngology COVID-19 Anosmia reporting tool [2]
SSTQ	Anosmia (onset in relation to other symptoms)	Smell	Spinato et al. [3]
SSTQ	Dysgeusia (Y/N)	Taste	Self-devised
SSTQ	Dysgeusia (when?)	Taste	American Academy of Otolaryngology COVID-19 Anosmia reporting tool [2]
SSTQ	Dysgeusia (onset in relation to other symptoms)	Taste	Spinato et al. [3]
SSTQ	Other symptoms (before smell or taste loss)	COVID-19	Self-devised
SSTQ	Symptoms (list)	COVID-19	Columbia University Irving Medical Centre COVID-19 symptom assessment/ GCCR [1]
SSTQ	Symptoms (at the time of smell/taste loss)	COVID-19	Columbia University Irving Medical Centre COVID-19 symptom assessment/ GCCR [1]

SSTQ	Problems with ability to smell	Smell	Smell Change Status Check (SCSC) [4,5]
SSTQ	Duration of problem with ability to smell	Smell	Smell Change Status Check (SCSC) [4,5]
SSTQ	Ratings of ability to smell before/during/after illness	Smell	GCCR [1]
SSTQ	Blockage of nose before/during/after illness	Smell	GCCR [1]
SSTQ	Fluctuations in sense of smell	Smell	GCCR [1]
SSTQ	Sense of smell since illness	Smell	GCCR [1]
SSTQ	Rated sense of smell	Smell	Self-devised
SSTQ	Problems with ability to taste	Taste	Smell Change Status Check (SCSC) [4,5]
SSTQ	Duration of problem with ability to taste	Taste	Smell Change Status Check (SCSC) [4,5]
SSTQ	Ratings of ability to taste before/during/after illness	Taste	GCCR [1]
SSTQ	Any other changes to taste (Y/N)	Taste	GCCR [1]
SSTQ	Tastes affected	Taste	GCCR [1]
SSTQ	Rated sense of Taste	Taste	Self-devised
SSTQ	Appetite	Eating behaviour and appetite Qs	Modified Monell-Jefferson Taste & Smell Questionnaire, Section 3 (Nutritional Information) [6]
SSTQ	Enjoyment of Food	Eating behaviour and appetite Qs	Modified Monell-Jefferson Taste & Smell Questionnaire, Section 3 (Nutritional Information) [6]
SSTQ	Effect on eating behaviours	Eating behaviour and appetite Qs	Modified Monell-Jefferson Taste & Smell Questionnaire, Section 3 (Nutritional Information) [6]
SSTQ	Altered salt/sugar/spice added to food	Eating behaviour and appetite Qs	Modified Monell-Jefferson Taste & Smell Questionnaire, Section 3 (Nutritional Information) [6]
SSTQ	Changes in liking	Eating behaviour and appetite Qs	Modified Monell-Jefferson Taste & Smell Questionnaire, Section 3 (Nutritional Information) [6]
SSTQ	Changes in craving	Eating behaviour and appetite Qs	Modified Monell-Jefferson Taste & Smell Questionnaire, Section 3 (Nutritional Information) [6]
SSTQ	Nauseated (Y/N)	Eating behaviour and appetite Qs	Modified Monell-Jefferson Taste & Smell Questionnaire, Section 3 (Nutritional Information) [6]
SSTQ	Nauseated (severity)	Eating behaviour and appetite Qs	Modified Monell-Jefferson Taste & Smell Questionnaire, Section 3 (Nutritional Information) [6]

SSTQ	Weight changes	Eating behaviour and appetite Qs	Modified Monell-Jefferson Taste & Smell Questionnaire, Section 3 (Nutritional Information) [6]
SSTQ	Weight and smell/taste problem	Eating behaviour and appetite Qs	Modified Monell-Jefferson Taste & Smell Questionnaire, Section 3 (Nutritional Information) [6]
SSTQ	Self-reported weight and height	Eating behaviour and appetite Qs	Self-devised
SSTQ	Loss of smell/taste and eating related QoL (matrix)	Eating behaviour and appetite Qs	Self-devised
SSTQ	Additional comments (free text response)	General	Self-devised
SSTQ	Symptoms (last 24 hours)	COVID-19	Columbia University Irving Medical Centre COVID-19 symptom assessment/ GCCR [1]
SNOT-22	Symptoms and social/emotional consequences of nasal disorder	Smell	Hopkins et al. [7]
HUT	Sense of smell (improvement)	Smell	Self-devised
HUT	Sense of smell (rating)	Smell	Self-devised
HUT	Sense of taste (improvement)	Taste	Self-devised
HUT	Sense of taste (rating)	Taste	Self-devised
HUT	Odours (Category 1) (absent/normal/heightened) [b]	Smell	Weizmann Smell tracker [8]
HUT	Odours (Category 2) (absent/normal/heightened) [b]	Smell	Weizmann Smell tracker [8]
HUT	Odours (Category 3) (absent/normal/heightened) [b]	Smell	Weizmann Smell tracker [8]
HUT	Odours (Category 4) (absent/normal/heightened) [b]	Smell	Weizmann Smell tracker [8]
HUT	Odours (Category 5) (absent/normal/heightened) [b]	Smell	Weizmann Smell tracker [8]
HUT	Odours (Category 6) (absent/normal/heightened) [b]	Smell	Weizmann Smell tracker [8]
HUT	Foods I cannot smell/taste	Smell/Taste	Modified Monell-Jefferson Taste & Smell Questionnaire, Section 1 [6]
HUT	Smell test (2 odour pens)	Smell	[Similar to testing procedure/questions used by]: Yale Smell Loss Survey [9]
HUT	Taste test (4 items)	Taste	[Similar to testing procedure/questions used by]: Yale Smell Loss Survey [9]

HUT [c]	Appetite	Eating behaviour and appetite Qs	Modified Monell-Jefferson Taste & Smell Questionnaire, Section 3 (Nutritional Information) [6]
HUT [c]	Enjoyment of Food	Eating behaviour and appetite Qs	Modified Monell-Jefferson Taste & Smell Questionnaire, Section 3 (Nutritional Information) [6]
HUT [c]	Effect on eating behaviours	Eating behaviour and appetite Qs	Modified Monell-Jefferson Taste & Smell Questionnaire, Section 3 (Nutritional Information) [6]
HUT [c]	Altered salt/sugar/spice added to food	Eating behaviour and appetite Qs	Modified Monell-Jefferson Taste & Smell Questionnaire, Section 3 (Nutritional Information) [6]
HUT [c]	Changes in liking	Eating behaviour and appetite Qs	Modified Monell-Jefferson Taste & Smell Questionnaire, Section 3 (Nutritional Information) [6]
HUT [c]	Changes in craving	Eating behaviour and appetite Qs	Modified Monell-Jefferson Taste & Smell Questionnaire, Section 3 (Nutritional Information) [6]
HUT [c]	Nauseated (Y/N)	Eating behaviour and appetite Qs	Modified Monell-Jefferson Taste & Smell Questionnaire, Section 3 (Nutritional Information) [6]
HUT [c]	Nauseated (severity)	Eating behaviour and appetite Qs	Modified Monell-Jefferson Taste & Smell Questionnaire, Section 3 (Nutritional Information) [6]
HUT [c]	Weight changes	Eating behaviour and appetite Qs	Modified Monell-Jefferson Taste & Smell Questionnaire, Section 3 (Nutritional Information) [6]
HUT [c]	Weight and smell/taste problem	Eating behaviour and appetite Qs	Modified Monell-Jefferson Taste & Smell Questionnaire, Section 3 (Nutritional Information) [6]
HUT [c]	Self-reported weight and height	Eating behaviour and appetite Qs	Self-devised
HUT [c]	Loss of smell/taste and eating related QoL (matrix)	Eating behaviour and appetite Qs	Self-devised
HUT [c]	GP consulted (Y/N)	General	Self-devised
HUT [c]	Treatment for Smell/Taste (Y/N)	General	Self-devised
HUT [c]	Medication prescribed (Y/N)	General	Self-devised
HUT [c]	Advice from other platforms (Y/N)	General	Self-devised
HUT [c]	Additional comments about smell/taste (free text response)	General	Self-devised
HUT	Symptoms (last 24 hours)	COVID-19	Columbia University Irving Medical Centre COVID-19 symptom assessment/ GCCR [1]
HUT	Additional comments (free text response)	General	Self-devised

Abbreviations are Singapore Smell and Taste Questionnaire (SSTQ), 2-item Sino-Nasal Outcome Test (SNOT-22), and Home-Use Test (HUT). [a] Although both had a similar symptoms list, we favoured the wording of the GCCR, as we believe this would be more easily understood by laypersons (i.e. less medical jargon). [b] Note, we use this to assess participants' self-reported perception of this smell; they were not required to find this household item and smell it (as in Iravani et al. (2020)). Also, we changed some of the items from the original categories for household items that are relatively common in Singapore. [c] These questions were only included in the extended version of the home-use test, which participants answered weekly.

References

1. Global Consortium for Chemosensory Research. GCCR Survey. Published 2020. Accessed July 10, 2020. https://sites.google.com/view/gcchemosensr/

2. American Academy of Otolaryngology - Head and Neck Surgery. COVID-19 Anosmia Reporting Tool. Published 2020. Accessed November 13, 2020. https://www.entnet.org/content/reporting-tool-patients-anosmia-related-covid-19

3. Spinato G, Fabbris C, Polesel J. Alterations in Smell or Taste in Mildly Symptomatic OutpatientsWith SARS-CoV-2 Infection. *JAMA*. 2020;323(20):1-3. doi:10.1056/NEJMoa2005412

4. Greenberg MS, Gilbert AN. *The SMELL CHANGE STATUS CHECK (SCSC) A Rapid Verbal Screening Tool for Assessing Recent Change in Smell Function during COVID-19 Evaluations By*.; 2020.

5. NHANES. 2011-2012 Data Documentation, Codebook, and Frequencies: Taste & Smell (CSQ_G). Published 2013. Accessed July 9, 2020. https://wwwn.cdc.gov/Nchs/Nhanes/2011-2012/CSQ_G.htm

6. The Asthma Centre. Modified Monell-Jefferson Taste & Smell Questionnaire.

Published 2020. Accessed November 13, 2020. http://www.asthmacenter.com/wp-content/uploads/Monell-Jefferson-Taste-Smell-Questionnaire.pdf

7. Hopkins C, Gillett S, Slack R, Lund VJ, Browne JP. Psychometric validity of the 22-item Sinonasal Outcome Test. *Clin Otolaryngol*. 2009;34(5):447-454. doi:10.1111/j.1749-4486.2009.01995.x

8. Iravani B, Arshamian A, Ravia A, et al. Relationship Between Odor Intensity Estimates and COVID-19 Prevalence Prediction in a Swedish Population. *Chem Senses*. Published online 2020. doi:10.1093/chemse/bjaa034

9. Yale School of Medicine. Yale School of Medicine Jiffy Test of Smell Sensitivity. Published 2020. Accessed July 10, 2020. https://yalesurvey.ca1.qualtrics.com/jfe/form/SV_3rzfStiKuEvtQvb
